# Supplementary material for: Balancing mcr-1 expression and bacterial survival is a delicate equilibrium between essential cellular defence mechanisms
Source: Nat Commun. 2017 Dec 12;8:2054. doi: 10.1038/s41467-017-02149-0 (PMC5727292; doi:10.1038/s41467-017-02149-0)
Supplement: Supplementary file 1 — Supplementary Information [file 41467_2017_2149_MOESM1_ESM.pdf]

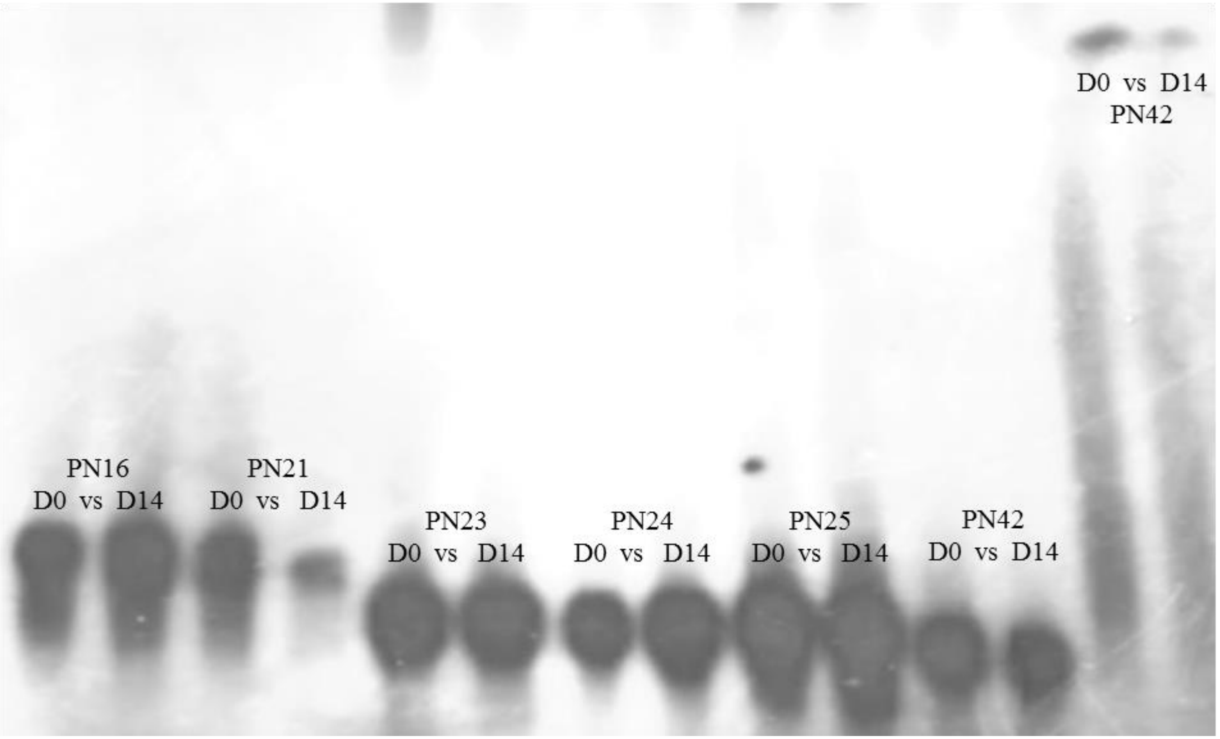


**Supplementary Fig. 1 In-gel hybridization with $^{32}$P-labeled *mcr-1* gene probe after PFGE of nuclease S1 digested genomic DNA.** Lanes from left to right are as followed: PN16 (D0 vs D14), PN21 (D0 vs D14), PN23 (D0 vs D14), PN24 (D0 vs D14), PN25 (D0 vs D14), PN42 (D0 vs D14) and PN43 (D0 vs D14).

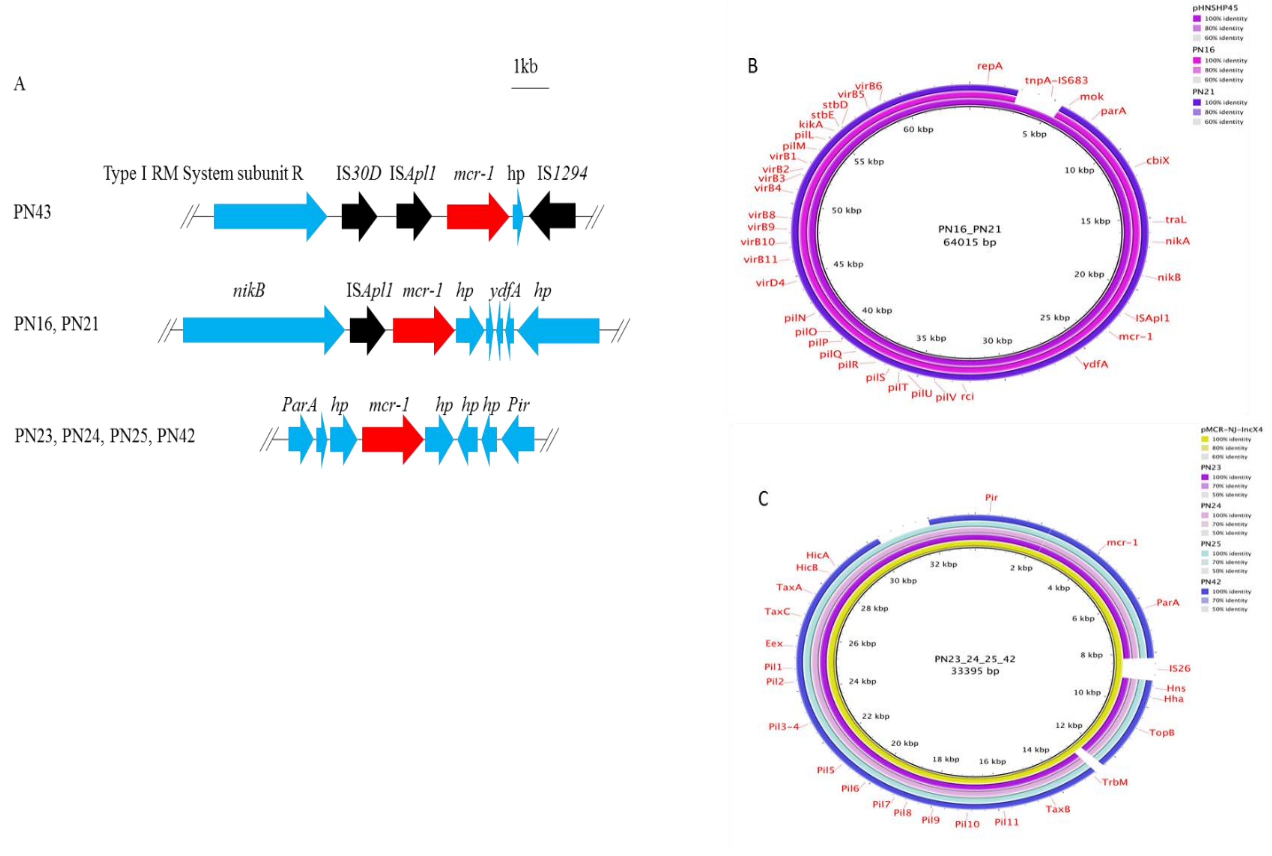


**Supplementary Fig. 2 Genetic contexts of *mcr-1* on plasmids and *E. coli* chromosome. A.** the flanking regions (approx.10kb) of *mcr-1*-carrying contigs from seven *E. coli* isolates from Thailand. The seven HLCRMs showed no amino acid mutations in *mcr-1*-carring contigs including their promoter and the immediate surrounding genetic contexts. The arrow indicated the ORFs and orientation of transcription, and the length of arrow represented the size of corresponding CDS. **B and C**, showed the complete genetic structure of plasmids from isolates PN16, PN21, PN23, PN24, PN25, and PN42. '*hp*' indicates hypothesis protein.

*E. coli* TOP10::pBAD induced

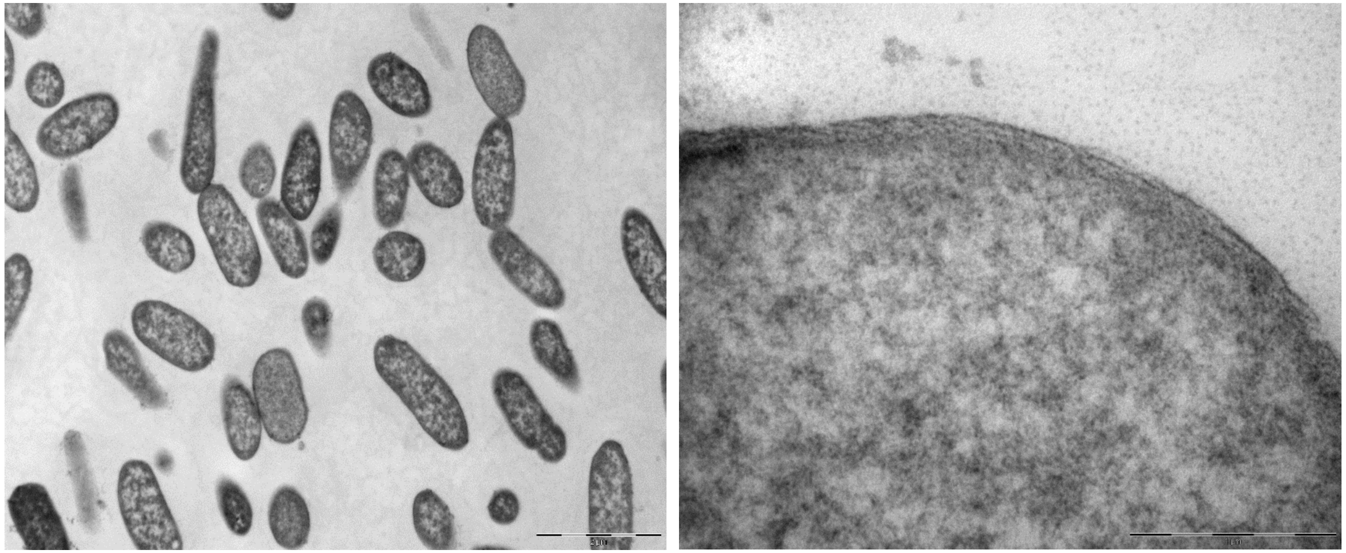

**Supplementary Fig. 3 TEM micrographs of control cells**: *E. coli* TOP10 with pBAD minus mcr-1 with L-arabinose induction (0.2%), cells are intact with a well-define inner and outer-membrane, and showed a highly homogeneous electron density in cytoplasm region. This result can rule out effects caused by high concentration of L-arabinose.

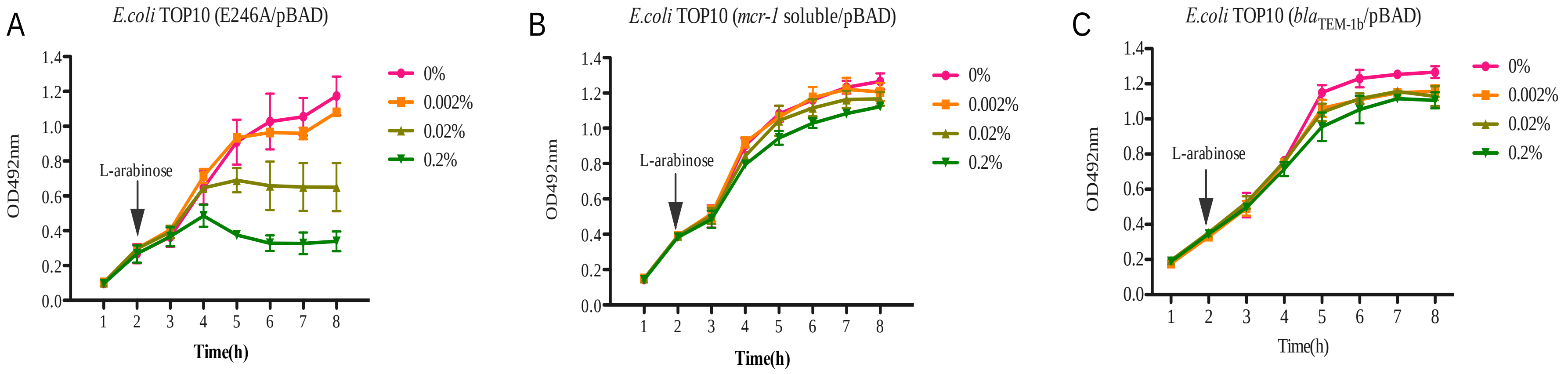


**Supplementary Fig. 4 *In vitro* growth curve of *mcr-1* derivatives and a negative control**. The means of three independent replicates were shown and the error bars represent the S.D (n=3).

A. *E.coli* TOP10 (E246A/pBAD) induced

B. *E.coli* TOP10 *mcr-1* soluble/pBAD induced

C. *E.coli* TOP10 (*bla*$_{TEM\text{-}1b}$/pBAD) induced

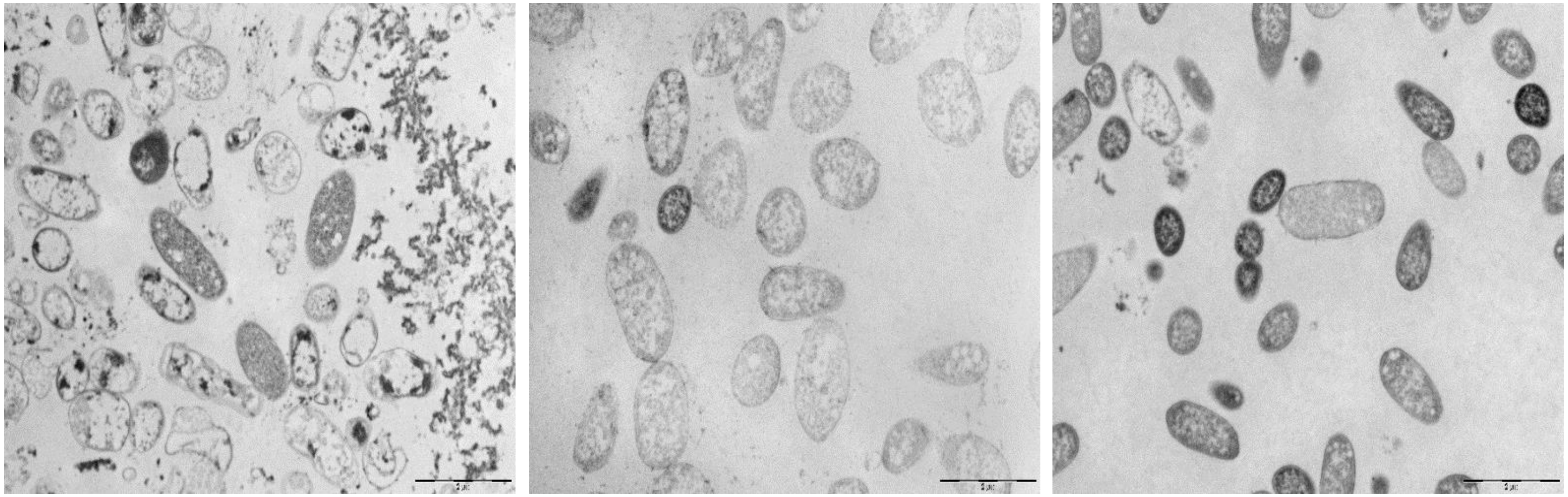

**Supplementary Fig. 5 TEM micrographs of *mcr-1* mutants.** A and B, TEM micrographs of *mcr-1* mutation implicated in its active site, E246A, and MCR-1 soluble domain, respectively. **C**, TEM micrographs of a negative control, *bla*$_{TEM\text{-}1b}$, a highly homogeneous electron density in cytoplasm region were observed.

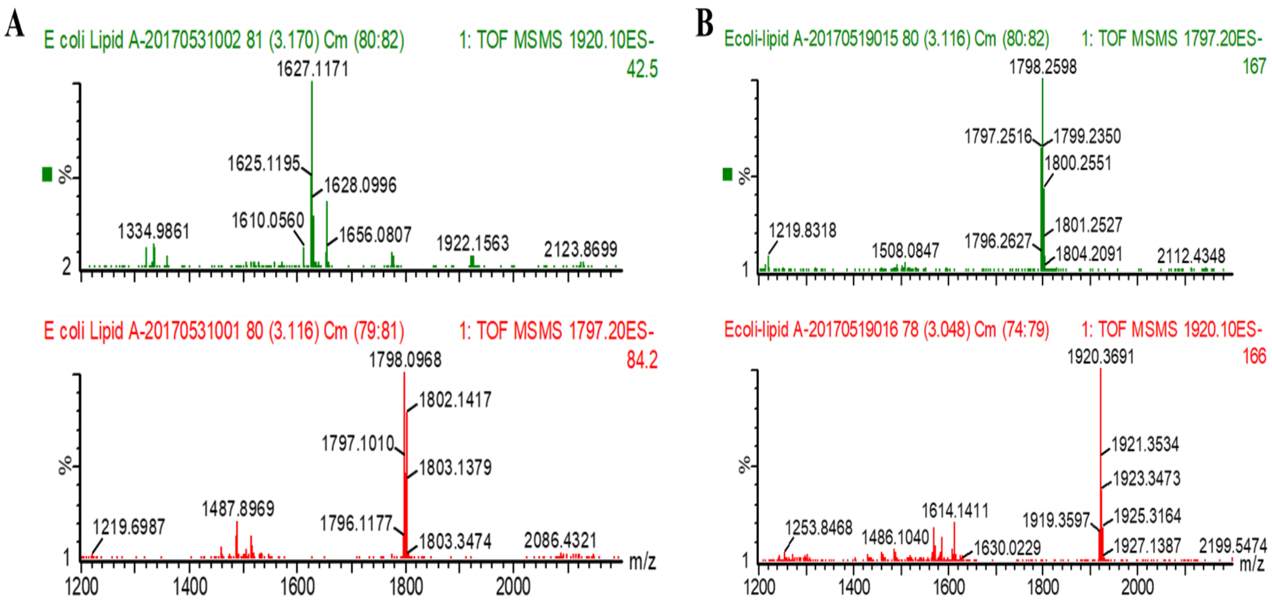


**Supplementary Fig. 6 ESI-QTOF/MS revealed the PEA modification of bacterial lipid A mediated by *MCR-1* gene.** A, ESI-MS/MS spectrum of the negative ion of the lipid A extracted from the constructed control strains *E. coli* W3110+ pUC19. The lipid A has a prominent peak at m/z 1797.10 and no PEA modification existed at m/z 1920. B, ESI-MS/MS spectrum visualization of the negative ion of the lipid A extracted from the constructed positive strains *E. coli* W3110 +pUC19-*mcr-1*. A PEA (123u) is added into the bis-phosphorylated hexaacylated lipid A (m/z 1920).

Note: In this study, the bacterial lipid A was analyzed using Waters Synapt HR mass spectrometry under two different ion channels (m/z 1797 and m/z 1920).

TOF MSMS 1920.10ES-: Selected ion monitoring of the product of lipid A modification at m/z 1920.10 with negative ion scan

TOF MSMS 1797.20ES-: Selected ion monitoring of the bacterial lipid A at m/z 1797.20 with negative ion scan

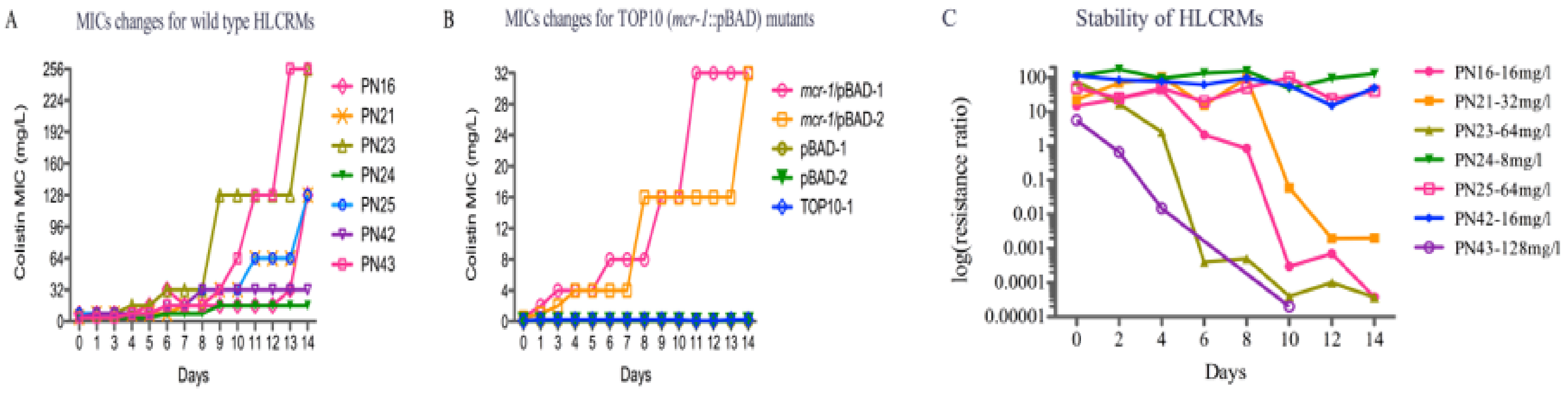


**Supplementary Fig. 7 Acquisition and stability of high-level colistin resistance mutants.** Changes in colistin minimum inhibitory concentrations (MICs) during 14-day challenging with increasing concentrations of colistin, in seven wild type MCRPEC (**A**) and three laboratory strains (**B**). **C**, the recovery of colistin resistance phenotype in high-level colistin mutants after 14-day passage in colistin-free medium. The resistance ratios represent the ratio between colony-forming units (CFUs) of colistin resistance cells and the total CFUs including resistant strains and susceptible strains.

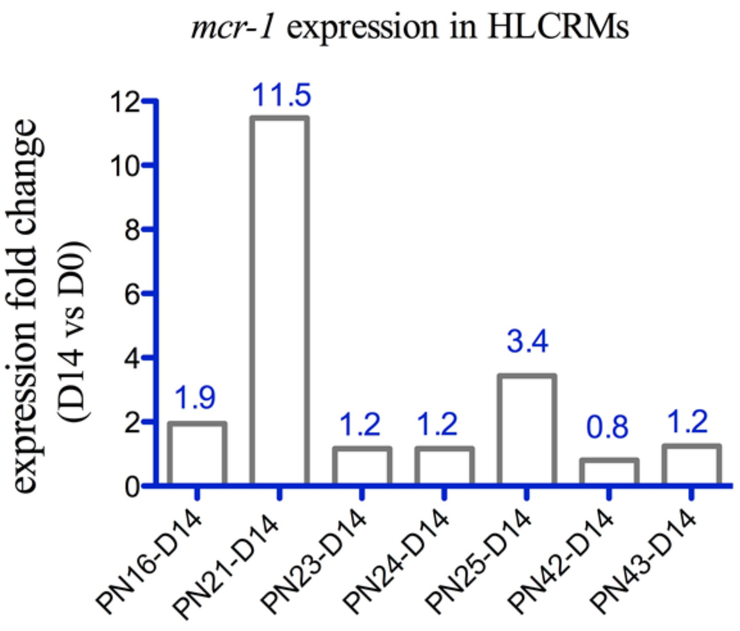


**Supplementary Fig. 8 Expression changes of *mcr-1* gene in HLCRMs mutants, comparing to their parental strains.** Fold changes of *mcr-1* expression were obtained by the ΔΔCT analysis method using mean CT value (n=2).

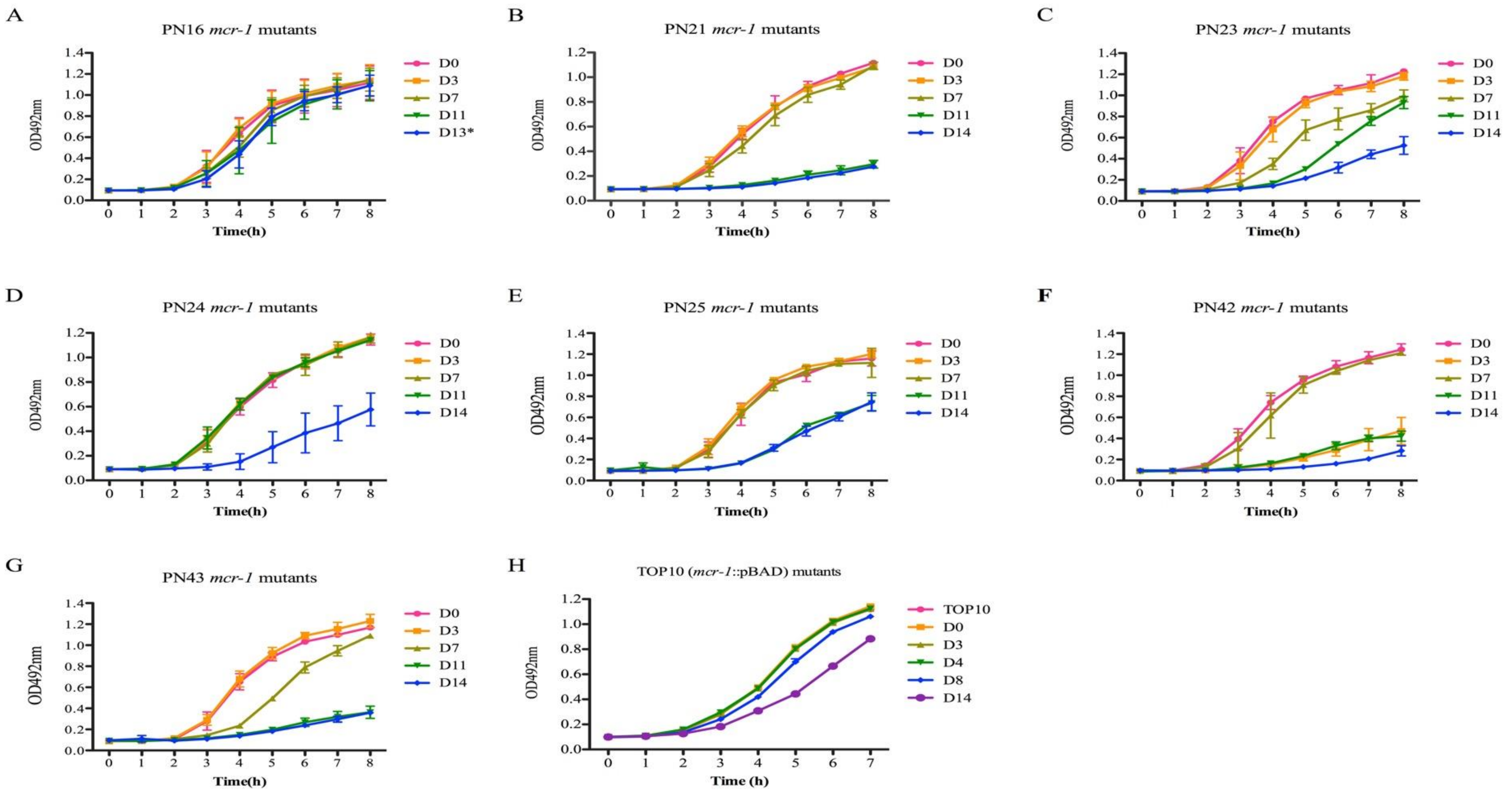


**Supplementary Fig. 9 *In vitro* growth curve of seven *mcr-1* positive strains and their derivatives. A-G**, indicated the growth curves of seven WT strains (PN16, 21, 23, 24, 25, 42 and 43) and their derivatives (samples from serial time points: D0, D3, D7, D11 and D14). **H**, indicated the growth rate of *E.coli* TOP10 (*mcr-1*::pBAD) and their derivatives (samples from serial time points: D0, D3, D4, D8 and D14). The $OD_{492}$ value was determined at every one hour. The means of three independent replicates were shown and the error bars represent the S.D (n=3). * for strain PN16, its day-14 mutant are unable to grow, so we tested day-13 mutant instead.

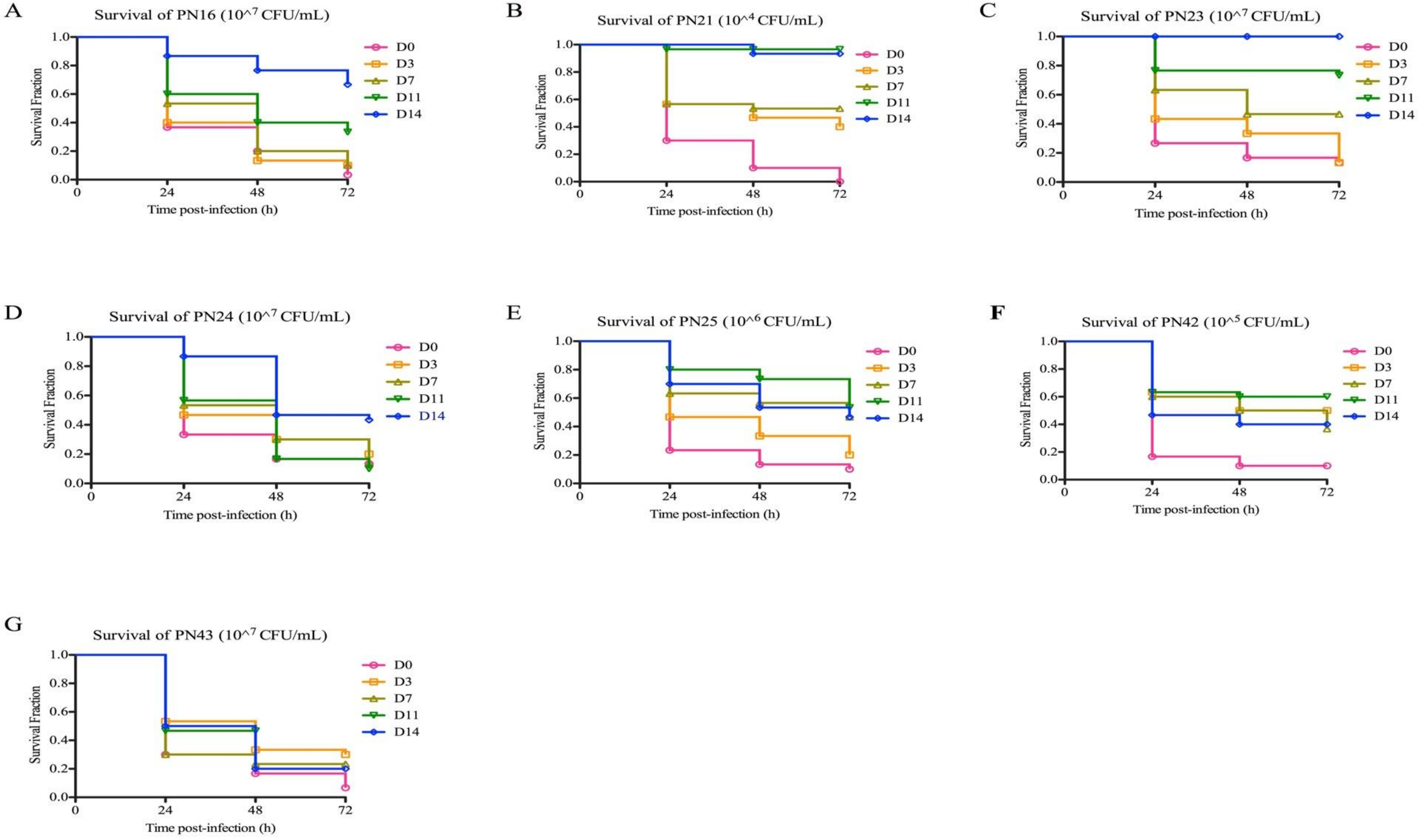


**Supplementary Fig. 10 individual *Galleria Mellonella* killing models. A-G**, indicated *G. Mellonella* killing curves in seven WT strains (PN16, 21, 23, 24, 25, 42 and 43) and their respective mutants with series time points D0, D3, D7, D11 and D14.

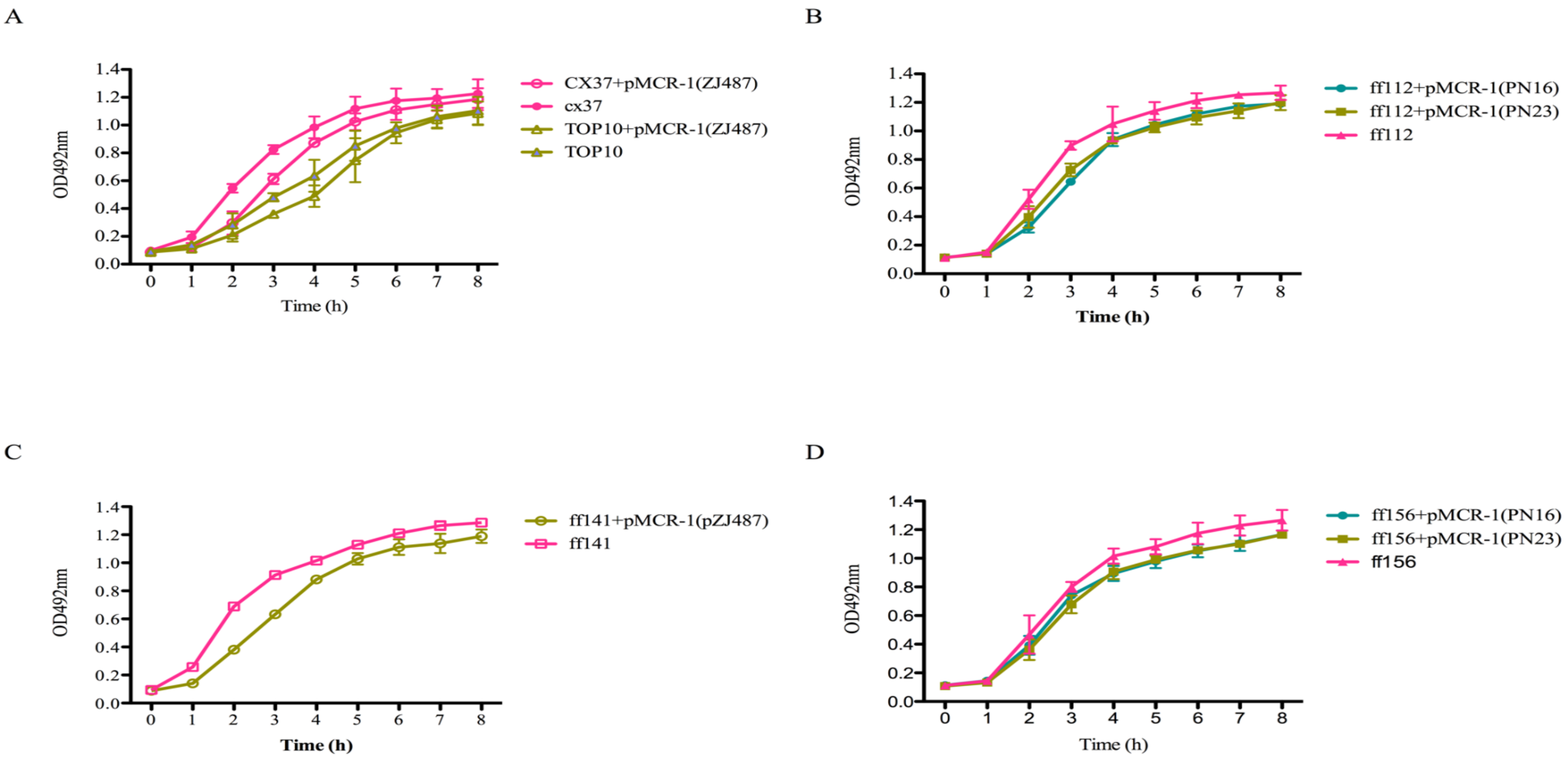


**Supplementary Fig. 11 *In vitro* growth curve of six *mcr-1* positive E.coli (MCRPEC) and their counterpart non-MCRPEC.** The means of three independent replicates were shown and the error bars represent the S.D (n=3).

| Supplementary Table 1 Characteristics of MCR-1 carrying bacteria(MCRPEC) and non-MCRPEC | | | | | | | | | |
|---|---|---|---|---|---|---|---|---|---|
| **No.** | **Date of isolation** | **Source** | **Colistin MICs(mg/l)** | **MLST*** | ***mcr-1* location** | **Plasmid (type/size)** | *mcr-1* copy number | **Additional resistance gene** | **reference** |
| **PN16** | 27/10/2013 | chicken meat | 8 | ST-2040 | plasmid | IncI2 /60,488 bp | 3.1 | *aadA1,blaTEM-1b, blaCMY-2, QnrS1, cmlA1, Sul3, Tet(M)* | This study |
| **PN21** | 12/02/2014 | chicken faeces | 4 | ST-24** | plasmid | IncI2 /60,989bp | 2.1 | *acc(3)-Iid, blaTEm-1A, blaCTX-M-55, QnrS1, fosA, tetA* | This study |
| **PN23** | 22/02/2014 | duck faeces | 8 | ST-1121 | plasmid | IncX4 /33,858bp | 5.6 | *tet(B)* | This study |
| **PN24** | 22/02/2014 | duck faeces | 4 | ST-3631 | plasmid | IncX4 /35,075bp | 4.3 | *aadA1, adc(3)-Iid, aadA2, blaTEM-1B, blaCTX-M-55, QnrS1, mef(B), catA2, cmlA1, sulA2, tet(A), dfrA12* | This study |
| **PN25** | 22/02/2014 | duck faeces | 8 | ST-101 | plasmid | IncX4 /34,117bp | 1.8 | *blaCTX-M-14, tet(B)* | This study |
| **PN42** | 15/11/2013 | faeces from healthy human | 4 | ST-744 | plasmid | IncX4 /32,995bp | 3.2 | *aph(3')-la, strA, strB, aadA1, acc(3)-Iid, aadA5, aadA2, blaCTX-M-55, blaCTX-M-14, blaTEM-1B, QnrS1, Mph(A), mef(B),catA1, cmlA1, sul2, sul3, sul1, tet(B), dffA17, dfrA12* | This study |
| **PN43** | 15/11/2013 | faeces from healthy human | 4 | ST-410 | chromosome | NA | 1 | *aadA2,aadA1, aac(3)-11d, blaTEM-IIB, blaCTX-M-55, QnrSI, inu(F), cmlA1, catA2, floR, sul2, sul3, tetA, tetM, dfrA12* | This study |
| **ff112#** | - | Human | 0.5 | ST-638 | - | - | - | - | This study |
| **ff141#** | - | Human | 0.5 | ST-589 | - | - | - | - | This study |
| **ff156#** | - | Human | 0.5 | ST-127 | - | - | - | - | This study |
| **CX37** | - | Human | 0.5 | ST-648 | - | - | - | - | [1] |

* WGS MLST was determined using CGE pipeline (https://cge.cbs.dtu.dk//services/MLST/) following Wirth et al. scheme[2] for all isolates except PN21. ** Jaureguy et al. scheme[3] was used to identify MLST type of strain PN21. # indicates that strains ff112, ff141 and ff156 are a group of wholly sensitive/susceptible *E. coli*, provided by Specialist Antimicrobial Chemotherapy Unit (SACU), Public Health Wales.

| **Supplementary Table2 Laboratory strains and plasmids used in this study** | | | | |
|---|---|---|---|---|
| **Plasmid name** | Host strain | Plasmid (size) | Colistin MIC (mg/L) | Reference |
| ***mcr-1*/pBAD** | *E.coli* TOP10 | ~6000bp | 2 | This study |
| **pBAD alone** | *E.coli* TOP10 | ~4100bp | 0.25 | Thermo Fisher, UK |
| ***E.coli* TOP10** | - | - | 0.25 | Thermo Fisher, UK |
| ***mcr-1*/pSU18** | *E.coli* TOP10 | ~4000bp | 4 | [4] |
| **GFP-pHT315** | DH5 *alpha* | - | 0.5 | [5] |
| ***bla*$_{TEM-1b}$/pBAD** | *E.coli* TOP10 | ~5000bp | 0.25 | This study |
| **E246A/pBAD** | *E.coli* TOP10 | ~6000bp | 0.25 | This study |
| ***mcr-1* soluble domain /pBAD** | *E.coli* TOP10 | ~5400bp | 0.25 | This study |

| Supplementary Table3 Primers and probes used in this study | | | |
|---|---|---|---|
| **Name** | sequencing(5'-3') | size (bp) | Reference |
| ***mcr-1* F** | GCTACTGATCACCACGCTGT | 953 | This study |
| ***mcr-1* R** | TGGCAGCGACAAAGTCATCT | | |
| ***16S* qF** | CATTGA CGTTACCCGCAGAA | 100 | [6] |
| ***16S* qF** | CGCTTTACGCCCAGTAATTCC | | |
| ***16S* probe** | FAM-CGTGCCAGCAGCCGCGGTA-TAMRA | | |
| ***rpoB*-qF3** | TCCTTTCTATCCAGCTTGACTCGT | 200 | [7] |
| ***rpoB*-qR3** | CGCAGTTTAACGCGCAGCGG | | |
| ***rpoB*-probe** | HEX-ACGTCAGCTACCGCCTTGGCGAACCGGTGT-BHQ1 | | |
| ***mcr-1* qF** | TGGCGTTCAGCAGTCATTAT | | [8] |
| ***mcr-1* qR** | AGCTTACCCACCGAGTAGAT | | |
| ***mcr-1* probe** | ROX-AGTTTCTTTCGCGTGCATAAGCCG- BHQ1 | | Modified with Dye and quencher |
| ***mcr-1* soluble F** | GGGGTACCACCATTTATCACGCCAAAGACG | 969 | This study |
| ***mcr-1* soluble R** | CGGAATTCGCGGATGAATGCGGTGCG | | |
| ***$bla_{TEM-1b}$* F** | GGGGTACC ATGAGTATTCAACATTTTCGTGTCG | 861 | This study |
| ***$bla_{TEM-1b}$* R** | CGGAATTC TTACCAATGCTTAATCAGTGAGGC | | |

**Supplementary Table4 MICs of colistin for *mcr-1*/pBAD after 14-day serial passage**

| **Passage** | MICs of Colistin (mg/L) | | | | |
|---|---|---|---|---|---|
| **days** | **mcr-1(1)** | **mcr-1(2)** | **pBAD-1** | **pBAD-2** | **TOP10** |
| **1** | 0.5 | 0.5 | 0.125 | 0.125 | 0.125 |
| **2** | 2 | 1 | 0.125 | 0.125 | 0.25 |
| **3** | 4 | 2 | 0.125 | 0.125 | 0.25 |
| **4** | 4 | 4 | 0.125 | 0.125 | 0.25 |
| **5** | 4 | 4 | 0.125 | 0.125 | 0.25 |
| **6** | 8 | 4 | 0.125 | 0.125 | 0.25 |
| **7** | 8 | 4 | 0.125 | 0.125 | 0.25 |
| **8** | 8 | 16 | 0.125 | 0.125 | 0.25 |
| **9** | 16 | 16 | 0.125 | 0.125 | 0.25 |
| **10** | 16 | 16 | 0.125 | 0.125 | 0.25 |
| **11** | 32 | 16 | 0.06 | 0.06 | 0.125 |
| **12** | 32 | 16 | 0.06 | 0.06 | 0.125 |
| **13** | 32 | 16 | 0.06 | 0.06 | 0.25 |
| **14** | 32 | 32 | 0.06 | 0.125 | 0.25 |

| **Supplementary Table 5 MICs of Colistin for seven wild type *mcr-1*-positive strains after 14-day serial passage** | | | | | | | |
|---|---|---|---|---|---|---|---|
| **Passage** | MICs of Colistin (mg/L) | | | | | | |
| **days** | **PN16** | **PN21** | **PN23** | **PN24** | **PN25** | **PN41** | **PN43** |
| **0** | 8 | 4 | 8 | 4 | 8 | 4 | 4 |
| **1** | 8 | 8 | 8 | 4 | 8 | 8 | 4 |
| **3** | 8 | 8 | 8 | 4 | 8 | 8 | 4 |
| **4** | 8 | 8 | 16 | 4 | 8 | 8 | 8 |
| **5** | 16 | 8 | 16 | 4 | 8 | 8 | 8 |
| **6** | 32 | 8 | 32 | 8 | 16 | 16 | 16 |
| **7** | 16 | 16 | 32 | 8 | 16 | 16 | 16 |
| **8** | 16 | 16 | 32 | 8 | 32 | 32 | 16 |
| **9** | 16 | 32 | 128 | 16 | 32 | 32 | 32 |
| **10** | 16 | 32 | 128 | 16 | 32 | 32 | 64 |
| **11** | 16 | 64 | 128 | 16 | 64 | 32 | 128 |
| **12** | 16 | 64 | 128 | 16 | 64 | 32 | 128 |
| **13** | 32 | 64 | 128 | 16 | 64 | 32 | 256 |
| **14** | 128 | 128 | 256 | 16 | 128 | 32 | 256 |

| **Supplementary Table 6 Relative Fitness (RF)** | | | | | **Mann-Whitney test** |
|---|---|---|---|---|---|
| RF(compared to the *mcr-1*/pBAD D0 Strain) | repeats | mean | SD | propagated errors | *p*-value |
| *E.coli* TOP10 | 6 | 1.0960 | 0.0129 | 0.0171 | 0.132 |
| *E.coli* TOP10 pBAD | 6 | 1.0955 | 0.0054 | 0.0134 | 0.0649 |
| *E.coli* TOP10 *mcr-1*/pBAD D3 | 6 | 1.0080 | 0.0062 | 0.0140 | 0.041* |
| *E.coli* TOP10 *mcr-1*/pBAD D4 | 6 | 1.0132 | 0.0257 | 0.0283 | 0.3095 |
| *E.coli* TOP10 *mcr-1*/pBAD D8 | 6 | 0.8258 | 0.0088 | 0.0165 | 0.0022** |
| *E.coli* TOP10 *mcr-1*/pBAD D14 | 6 | 0.7922 | 0.0084 | 0.0164 | 0.0022** |
| | | | | | |
| RF(compared to each corresponding parent) | | | | | |
| PN16-D14 | 6 | 0.9380 | 0.0809 | 0.0875 | 0.132 |
| PN21-D14 | 6 | 0.4114 | 0.0293 | 0.0816 | 0.0022** |
| PN23-D14 | 6 | 0.4083 | 0.0385 | 0.1404 | 0.0022** |
| PN24-D14 | 6 | 0.4382 | 0.0133 | 0.0445 | 0.0022** |
| PN25-D14 | 6 | 0.6047 | 0.0557 | 0.1196 | 0.0022** |
| PN42-D14 | 6 | 0.5169 | 0.0309 | 0.0650 | 0.0022** |
| PN43-D14 | 6 | 0.7779 | 0.0534 | 0.1003 | 0.0022** |
| | | | | | |
| RF(compared to *mcr-1*/pBAD uninduced strain – 0%#) | | | | | |
| *E.coli* TOP10 | 6 | 1.0000 | 0.0147 | 0.0177 | 0.8182 |
| *E.coli* TOP10 *mcr-1*/pBAD +0.0002% | 6 | 0.9992 | 0.0088 | 0.0132 | 0.6991 |
| *E.coli* TOP10 *mcr-1*/pBAD +0.002% | 6 | 0.9991 | 0.0153 | 0.0182 | 1 |
| *E.coli* TOP10 *mcr-1*/pBAD +0.02% | 6 | 0.7278 | 0.0031 | 0.0108 | 0.0022** |
| *E.coli* TOP10 *mcr-1*/pBAD +0.2% | 6 | 0.4272 | 0.0074 | 0.0262 | 0.0022** |

# indicates the concentration of L-arabinose (w/v). * indicates 0.01< *p* value <0.05 and ** indicates *p* value <0.01

**Supplementary Table 7 Antibiotic sensitivity profiles (mg/l) of MCRPEC and their respective HLCRMs**

| **Strains** | | CLT | CIP | ETP | CTX | AMK | TBM | GEN | NTF | CHL | TIG | FOS | VAN | AMP |
|---|---|---|---|---|---|---|---|---|---|---|---|---|---|---|
| **PN16** | Day 0 | 8 | 1 | 0.06 | 16 | 2 | 1 | 16 | 16 | 32 | 1 | 4 | 512 | 512 |
| | Day13* | 128 | 0.25 | 0.25 | 16 | 2 | 1 | 1 | 8 | 16 | 0.5 | 8 | 512 | 512 |
| **PN21** | Day 0 | 4 | 4 | 0.06 | 64 | 2 | 8 | 128 | 16 | 32 | 8 | 256 | 512 | 512 |
| | Day14 | 64 | 0.5 | 0.015 | 64 | 8 | 8 | 128 | 8 | 2 | 0.25 | 256 | 256 | 512 |
| **PN23** | Day 0 | 8 | 0.25 | 0.06 | 4 | 2 | 1 | 8 | 16 | 32 | 1 | 256 | 512 | 512 |
| | Day14 | 256 | 0.03 | 0.008 | 0.006 | 8 | 2 | 4 | 16 | 2 | 0.5 | 4 | 512 | 32 |
| **PN24** | Day 0 | 4 | 1 | 0.015 | 64 | 2 | 8 | 256 | 16 | 128 | 8 | 1 | 512 | 512 |
| | Day14 | 16 | 1 | 0.03 | 64 | 16 | 32 | 256 | 16 | 128 | 1 | 0.5 | 512 | 512 |
| **PN25** | Day 0 | 8 | 0.008 | 0.015 | 4 | 2 | 1 | 1 | 8 | 4 | 1 | 1 | 256 | 512 |
| | Day14 | 128 | 0.015 | 0.008 | 4 | 2 | 0.5 | 1 | 8 | 2 | 0.5 | 1 | 256 | 512 |
| **PN42** | Day 0 | 4 | 32 | 0.015 | 8 | 2 | 2 | 32 | 32 | 128 | 2 | 1 | 512 | 512 |
| | Day14 | 32 | 128 | 0.03 | 32 | 4 | 8 | 256 | 4 | 8 | 0.125 | 4 | 256 | 512 |
| **PN43** | Day 0 | 4 | 128 | 0.06 | 32 | 2 | 4 | 64 | 8 | 256 | 4 | 1 | 512 | 512 |
| | Day14 | 256 | 128 | 0.03 | 64 | 4 | 4 | 128 | 8 | 128 | 0.125 | 2 | 256 | 512 |

CLT, Colistin; CIP, Ciprofloxicin; ETP, Ertapenem; CTX, Cefotaxime; AMK, Amikacin; TBM, Tobramycin; GEN, Gentamicin; NTF, Nitrofurantoin; CHL, Chloramphenicol; TIG, Tigecycline; FOS, Fosfomycin; VAN, Vancomycin; AMP, Ampicillin. * for strain PN16, day-14 mutant are unable to grow, so we tested day-13 mutant instead.

**Supplementary References**


1. Wang Y, *et al.* Prevalence, risk factors, outcomes, and molecular epidemiology of mcr-1-positive Enterobacteriaceae in patients and healthy adults from China: an epidemiological and clinical study. *The Lancet Infectious Diseases* **17**, 390-399 (2017).

2. Wirth T, *et al.* Sex and virulence in Escherichia coli: an evolutionary perspective. *Molecular microbiology* **60**, 1136-1151 (2006).

3. Jaureguy F, *et al.* Phylogenetic and genomic diversity of human bacteremic Escherichia coli strains. *BMC genomics* **9**, 560 (2008).

4. Hinchliffe P, *et al.* Insights into the Mechanistic Basis of Plasmid-Mediated Colistin Resistance from Crystal Structures of the Catalytic Domain of MCR-1. *Sci Rep* **7**, 39392 (2017).

5. Daou N, *et al.* IlsA, a unique surface protein of Bacillus cereus required for iron acquisition from heme, hemoglobin and ferritin. *PLoS pathogens* **5**, e1000675 (2009).

6. Spano G, Beneduce L, Terzi V, Stanca AM, Massa S. Real-time PCR for the detection of Escherichia coli O157:H7 in dairy and cattle wastewater. *Letters in applied microbiology* **40**, 164-171 (2005).

7. Jones LS, *et al.* Characterization of plasmids in extensively drug-resistant acinetobacter strains isolated in India and Pakistan. *Antimicrob Agents Chemother* **59**, 923-929 (2015).

8. Irrgang A, *et al.* Prevalence of mcr-1 in E. coli from Livestock and Food in Germany, 2010-2015. *PloS one* **11**, e0159863 (2016).
